# Efficacy and safety of topical minocycline preparations for papulopustular rosacea: a systematic review and meta-analysis

**DOI:** 10.3389/fmed.2025.1517825

**Published:** 2025-04-01

**Authors:** Awadh Alamri, Abdulrahman H. Alsamadani, Rose A. Alraddadi, Mulham Kalantan, Randa Khafaji, Bader Bashrahil, Hassan Bogari, Athoub Kadasa, Abdulhadi Jfri

**Affiliations:** ^1^College of Medicine, King Saud Bin Abdulaziz University for Health Sciences, Jeddah, Saudi Arabia; ^2^Division of Dermatology, Department of Medicine, Ministry of the National Guard-Health Affairs, Jeddah, Saudi Arabia; ^3^King Abdullah International Medical Research Center, Jeddah, Saudi Arabia

**Keywords:** rosacea, topical, minocycline, foam, systematic review

## Abstract

**Background:**

Rosacea is a chronic inflammatory skin condition. Papulopustular rosacea (PPR), one of the subtypes of rosacea, presents with papules and pustules (Pelle, 2008). Topical minocycline allow the delivery of high concentrations of the medication to the skin while decreasing systemic exposure thereby evading side effects (Jones et al., 2021, Webster et al., 2020). This study aims to review the literature to delineate the efficacy and safety of topical preparations of minocycline in the treatment of moderate to severe papulopustular rosacea.

**Methods:**

This systematic review included randomized clinical trials (RCT) only that compared the efficacy and safety of 1.5% minocycline foam and 1%, 3% minocycline gel versus placebo in patients with moderate to severe papulopustular rosacea. We performed a systematic search in Medline, Cochrane Central Register of Controlled Trials (CENTRAL), Scopus, and ClinicalTrials.gov. Efficacy outcomes included the absolute change in inflammatory lesion counts, the percentage change in the inflammatory lesion count, the percentage of participants achieving Investigator Global Assessment (IGA) with improvement of at least two grades, and the proportion of individuals that achieved an IGA 0/1 score (“clear” or “almost clear”). This paper was conducted in adherence to PRISMA guidelines. Also, we have registered our protocol in PROSPERO (CRD42023447486). Quality assessment of the included studies was conducted using ROB-2 tool. Additionally, we have assessed the level of evidence using GRADE too. The analysis was performed using RevMan.

**Results:**

Five randomized controlled trials with low risk of bias were included in the quantitative synthesis with a total of 2,453 enrolled participants. Minocycline (FMX103) 1.5% foam yielded statistically significant results in terms of IGA score indicating treatment success [Risk Ratio (RR) = 1.31, 95% confidence interval (CI) = 1.04–1.66, *P* = 0.02]. FMX103 and minocycline gel 1% and 3% had significant results in absolute change in inflammatory lesion count (RR = 3.49, 95% CI = 2.61–4.36, *P* < 0.00001). Change in inflammatory lesion count from baseline with minocycline 1.5% foam was significantly reduced (RR = 9.45, 95% CI = 5.84–13.06, *P* < 0.00001). Other indicators of symptom reduction were not significant for both foam and gel preparations.

**Conclusion:**

Our findings suggest that topical preparations of minocycline provide statistically significant results in reducing absolute inflammatory lesion count and having IGA treatment success among patients with moderate to severe papulopustular rosacea. Further studies, however, should assess the efficacy of different concentrations and combinations of minocycline to better delineate the effect of this drug in the clinical aspect.

**Systematic review registration:**

PROSPERO, identifier CRD42023447486, https://www.crd.york.ac.uk/PROSPERO/view/CRD42023447486.

## Introduction

Rosacea is a chronic inflammatory skin condition that predominates across the centrofacial convexities including the cheeks, nose, chin, and the central forehead. The disease is characterized by a spectrum of symptoms which include erythema, phymatous alterations, papules, pustules, and telangiectasia (4). As rosacea can vary in its phenotypic presentation, it was further stratified into four subtypes by the National Rosacea Society (NRS). The subdivision encompasses papulopustular, erythematotelangiectatic, ocular, as well as phymatous rosacea. An additional variant, known as lupoid or granulomatous rosacea, was also recognized by the NRS (5, 6).

The pathogenesis of Rosacea is complex with an interplay of several factors. Evidently, neurovascular dysregulation coupled with abnormal innate immune system responses are accountable for the development of the disease’s characteristic erythema and telangiectasia. Also, increased activity of mast cells, plasma cells, and macrophages, in addition to Th1 and Th17 results in the formation of papules. Nonetheless, neutrophil-recruiting chemokines trigger the production of pustules (7, 8).

Papulopustular rosacea (PPR), one of the subtypes of rosacea, presents with unremitting Centro-facial erythema along with the eruption of papules and pustules (1). In addition to avoiding triggers and sun protection, treatment measures for PPR include a combination of topical and systemic treatments depending on severity. Systemic treatment is utilized in moderate to severe PPR commonly involves oral antibiotics, namely tetracyclines. Specifically, antibiotics are given at a sub-antimicrobial dosage to employ their anti-inflammatory effect. Minocycline, a tetracycline antibiotic, has proven highly effective in treating PPR, but adverse effects including morbilliform eruptions, photosensitivity, gastrointestinal discomfort, and liver disorders limit its long-term use (9, 10). Furthermore, multiple formulations of topical minocycline were developed and are available in either foam or gel preparations. Treatment of papulopustular rosacea often involves a combination of topical and oral therapies to manage inflammation, reduce lesions, and prevent flare-ups. Topical treatments, typically used for mild to moderate cases, include metronidazole, azelaic acid, ivermectin, and dapsone, which help control inflammatory lesions and target potential Demodex mite involvement (9). For moderate to severe cases or when topical treatments are insufficient, oral antibiotics such as doxycycline or minocycline are commonly prescribed due to their anti-inflammatory properties (11). In resistant cases, low-dose oral isotretinoin may be considered, along with advanced options like laser and light therapies to address persistent erythema (9, 11). Additionally, brimonidine or oxymetazoline can reduce facial redness by constricting blood vessels. Effective management requires a tailored approach based on individual patient factors and severity, combined with education on avoiding triggers such as sun exposure, spicy foods, and alcohol.

Topical formulations of minocycline allow the delivery of high concentrations of the medication to the skin while decreasing systemic exposure thereby evading systematic side effects (2, 3). This study aims to review the literature to delineate the efficacy and safety of topical preparations of minocycline in the treatment of moderate to severe papulopustular rosacea. By offering insights into a different therapeutic strategy that might yield good outcomes with potentially fewer systemic side effects than oral antibiotics, this research will advance the profession of dermatology. Furthermore, it might support medical professionals in making well-informed decisions about the treatment of papulopustular rosacea, which could enhance patient outcomes and quality of life.

## Methods

The studies were conducted following the methodology specified by the Cochrane Collaboration, with reporting guided by the Preferred Reporting Items for Systematic Reviews and Meta-Analyses (PRISMA) guidelines. Also, we have registered our protocol in PROSPERO (CRD42023447486) (12).

### Eligibility criteria

This systematic review included randomized clinical trials (RCTs) that compare the efficacy and safety of 1.5% minocycline foam and 1%, 3% minocycline gel versus placebo in patients with moderate to severe papulopustular rosacea. Eligible subjects were male or female aged ≥ 18 years who were diagnosed at least 6 months ago with moderate or severe papulopustular rosacea measured by Investigator’s Global Assessment (IGA) score. Individuals who used vitamin A supplements or oral retinoids within 6 months were excluded, as was the use of systemic antibiotics, systemic corticosteroids, or topical retinoids within 1 month, or the use of other topical medications (antibiotics, corticosteroids) within 2 weeks of randomization. Females who are in pregnancy, planning to become pregnant, or lactating were excluded, as were patients with skin conditions that could interfere with the assessment or diagnosis of rosacea.

### Search strategy

We performed a systematic search in Medline, Cochrane Central Register of Controlled Trials (CENTRAL), Scopus, and ClinicalTrials.gov for any data related to the use of minocycline 1.5% foam and 1%, 3% minocycline gel in the treatment of moderate to severe papulopustular rosacea. The search approach included appropriate keywords and mesh terms related to minocycline and rosacea (available in Supplementary materials).

### Study selection and data extraction

The studies were screened following the methodology outlined by the Cochrane Collaboration and adhered to PRISMA guidelines for reporting transparency. Eligibility criteria were applied during the selection process, and duplicates were removed after evaluating the titles and abstracts for relevance. For possibly eligible research, full-text papers were acquired, and their inclusion was further assessed. The articles that matched our eligibility criteria were reviewed and data on the trial characteristics, demographics of the participants, intervention, efficacy outcomes, and adverse events were extracted independently by two reviewers. A third author’s viewpoint was obtained after a thorough review of any discrepancies in the extracted data. Two reviewers extracted data from the included RCTs that matched our eligibility criteria.

### Outcomes

The choice of outcomes was based on the usually reported primary and secondary outcomes in clinical trials. Efficacy outcomes included the absolute change in inflammatory lesion counts, the percentage change in the inflammatory lesion count, the percentage of participants achieving IGA treatment success with improvement of at least two grades, and the proportion of individuals that achieved an IGA 0/1 score (“clear” or “almost clear”). Weeks 12, 40, and 52 were the time point of assessment for efficacy outcomes. Safety assessment included physical examination, vital signs, treatment-emergent adverse events (TEAEs), and laboratory investigation results. Local dermal tolerability assessment was also performed to measure the signs and symptoms, such as erythema, telangiectasia, burning/stinging, flushing/blushing, and dryness/xerosis.

### Meta-analysis

RevMan (Review Manager) version 5.3 (Cochrane Collaboration) was used for data analysis. For all statistical studies, the random-effects model was used. Statistical significance was defined as *p* < 0.05 with a 95% confidence level and I2 was used to assess the statistical heterogeneity. Change in inflammatory lesion count from baseline and absolute change in the inflammatory lesion count were the only continuous variables, and their effect sizes were measured using mean differences (MD). Data dispersion was calculated using standard deviation (SD). The dichotomous outcomes (IGA treatment success, dermal tolerability assessment) effect was presented as risk ratio (RR). Subgroup analysis was used to examine the effect of various regimens by classifying the data into three subgroups. The first subgroup included participants who were administered 1.5% minocycline foam once daily. The second and third subgroups included patients who were given 1% and 3% minocycline gel twice daily, respectively. The Grading of Recommendations Assessment, Development, and Evaluation (GRADE) criteria was used to assess the quality of evidence of outcomes.

### Risk of bias assessment

All authors independently analyzed the risk of bias for all studies included using The Revised Cochrane risk of bias tool. This instrument assesses various forms of bias, including blinding, allocation concealment, random sequence generation, partial outcome data, and selective outcome reporting.

## Results

**FIGURE 1:**
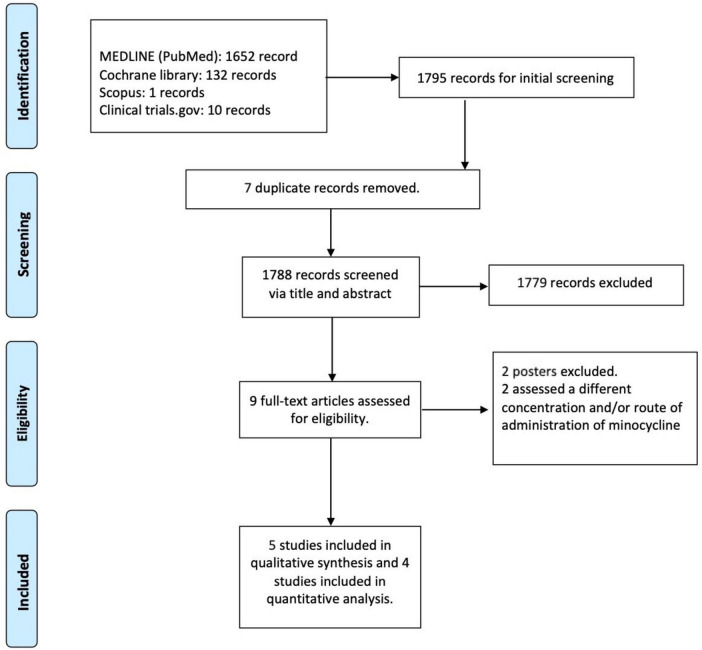
Flow chart of the selection process.

A total of 1,795 studies were identified through the database search. After screening titles and abstracts, 1,779 studies were excluded as they did not meet our eligibility criteria. Additionally, seven duplicate records and two poster presentations were removed. Following a full-text review, two more studies were excluded due to differences in minocycline concentrations and/or routes of administration. Ultimately, five randomized controlled trials (RCTs) met the criteria for qualitative assessment. Among these, four studies evaluated minocycline 1.5% foam, while one study examined minocycline 1% and 3% gel. Notably, one of the four studies on FMX103 (Gold et al., Study 13) was an extension study (Figure 1).

### Baseline characteristics

A total of 2,453 participants were enrolled in the included five RCTs, prescribing different minocycline regimens. The number of patients who were given minocycline 1.5% foam was 1,420, while minocycline gel 1% and 3% arms were 92 and 96, respectively. The placebo/vehicle foam arm included 845 participants in total. of 2,453 patients, the mean age ranges from 48.9 to 54.8 with females consisting of the majority with 70% (*n* = 1709), while males were 30% (*n* = 745) of the enrolled participants. Patients’ characteristics of the included RCTs are listed in Table 1. Data regarding the fund and endpoints for efficacy and safety were mentioned in Supplementary Table 2. Also, the results for each study were mentioned in Supplementary Tables 3, 4.

**TABLE 1:** Demographic data of included participants.

Study	Demographics											Treatment regimen		
	**Age**	**Gender**		**Race**					**Ethinicity**					
	**Mean**	**Female**	**Male**	**White**	**Black or African American**	**Asian**	**Others**	**Unknown or Not Reported**	**Hispanic or Latino**	**Not Hispanic or Latino**	**Unknown or Not Reported**	**Formulation**	**Frequency**	**Duration**
**NCT03142451, study 11**														
FMX103 1.5% foam: (*N* = 495)	48.9	355	140	474 (95.8%)	7 (1.4%)	6 (1.2%)	8 (1.6%)	0	165 (33.3%)	328 (66.3%)	2 (0.4%)	1.5% foam	Once daily	12 weeks
Vehicle: (*N* = 256)	49.7	186	70	241 (94.1%)	4 (1.6%)	6 (2.3%)	4 (1.6%)	1 (0.4%)	88 (34.4%)	168 (65.6%)	0			
**NCT03142451, study 12**														
FMX103 1.5% foam: (*N* = 514)	50.9	365	149	499 (97.1%)	7 (1.4%)	5 (1%)	2 (0.4%)	1 (0.2%)	166 (32.3%)	348 (67.7%)	0	1.5% foam	Once daily	12 weeks
Vehicle: (*N* = 257)	50.9	168	89	250 (97.3%)	1 (0.4%)	2 (0.8%)	3 (1.2%)	1 (0.4%)	86 (33.5%)	171 (66.5%)	0			
**NCT02601963**														
FMX103 1.5% foam: (*N* = 79)	51.2	53 (67.1)	26 (32.9)	98.7	N/A	N/A	1.3	N/A	N/A			1.5% foam	Once daily	12 weeks
Vehicle: (*N* = 78)	N/A	41(52.6)	37(47.4)	100			0							
**NCT03263273**														
(Minocycline 1% gel): (*N* = 92)	51.5	62 (67%)	30 (33%)	88 (96%)	2 (2%)	2 (2%)	N/A	N/A	28 (30%)	64 (70%)	N/A	1% gel	Twice daily	12 weeks
Vehicle: (*N* = 82)	52	56 (68%)	26 (32%)	80 (98%)	0	2 (2%)			19 (23%)	63 (77%)				
**NCT03263273**														
(Minocycline 3% gel): (*N* = 96)	50	71 (74%)	25 (26%)	94 (98%)	0	1 (1%)	1 (1%)	N/A	27 (28%)	69 (72%)	N/A	3% gel	Twice daily	12 weeks
Vehicle: (*N* = 82)	52	56 (68%)	26 (32%)	80 (98%)	0	2 (2%)	N/A		19 (23%)	63 (77%)				

### Risk of bias

**FIGURE 2:**
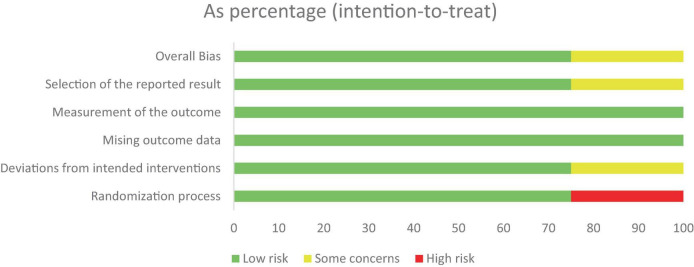
Weighted-bar plot of the risk of bias.

**FIGURE 3:**
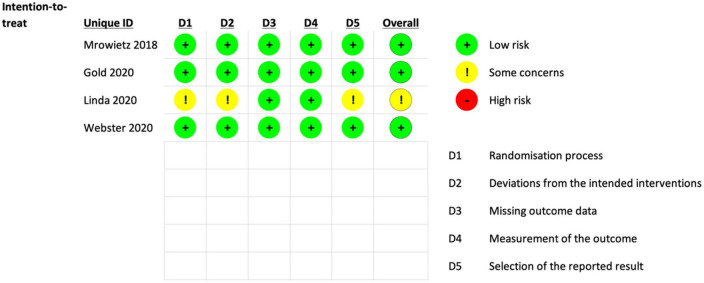
Traffic-light plot of the risk of bias.

Independently, all authors utilized the Revised Cochrane Risk of Bias tool to assess the quality of the included RCTs. Most studies showed a low risk of bias in all five domains except for the open-label extension study (study 13) Gold et al 2020 which has some concerns about the randomization process, deviations from the intended interventions, and selection of reported results (Figures 2, 3).

### Efficacy outcomes

**FIGURE 4:**
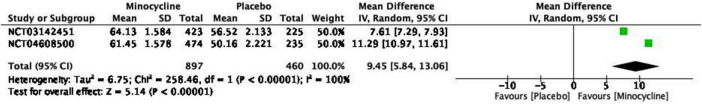
Change in inflammatory lesion count.

Of the included RCTs, only two studies of minocycline foam assessed the change in inflammatory lesion count from baseline to week 12. In both studies, minocycline 1.5% foam resulted in a significant change in inflammatory lesion count from baseline [MD = 9.45, 95% CI = (5.84,13.06), *P* < 0.00001, I2 = 100%] (Figure 4). GRADE assessment revealed that this outcome had a high certainty of evidence (Supplementary Table 1).

**FIGURE 5:**
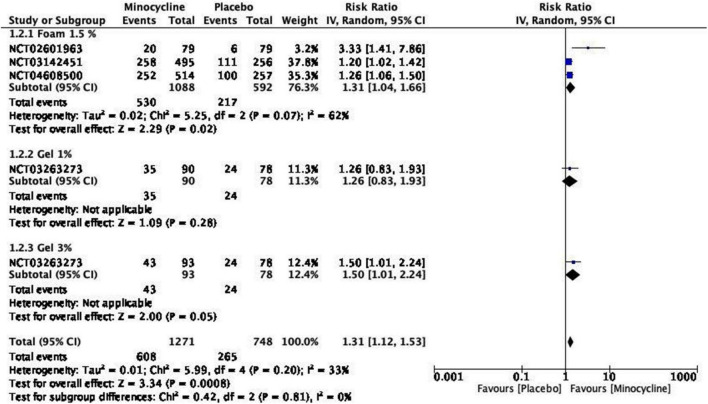
IGA treatment success.

Both minocycline foam 1.5% and gels 1%, 3% achieved significant improvement in IGA score assessment at week 12 [RR = 1.31, 95% CI = (1.12, 1.53), *P* = 0.0008, I2 = 33%]. Minocycline foam was significantly superior to both gel doses concerning IGA treatment success [RR = 1.31, 95% CI = (1.04, 1.66), *P* = 0.02, I2 = 62%]. The meta-analysis showed that minocycline gel 1% was insignificant to achieve success in IGA score [RR = 1.26, 95% CI = (0.83, 1.93), *P* = 0.28], while the 3% dose was at cut-off point [RR = 1.50, 95% CI = (1.01, 2.24), *P* = 0.05] (Figure 5). GRADE assessment revealed that this outcome had a high certainty of evidence (Supplementary Table 1).

As for the absolute change in inflammatory lesion count, three RCTs were included in the analysis. Minocycline foam and gel are considered significantly effective when compared to vehicle [MD = 3.49, 95% CI = (2.61,4.36), *P* < 0.00001, I2 = 37%]. Minocycline foam 1.5% demonstrated the most outstanding effect in comparison to vehicle [MD = 3.21, 95% CI = (1.64,4.77), *P* < 0.0001, I2 = 79%], while 1% gel [MD = 3.70, 95% CI = (1.31,6.09), *P* = 0.002] was superior to the 3% gel [MD = 4.20, 95% CI = (0.61,7.79), *P* = 0.02] (Supplementary Figure 1). GRADE assessment revealed that this outcome had a high certainty of evidence (Supplementary Table 1).

As for topical minocycline, various skin irritant reactions were presented. Participants of only two studies that assessed minocycline foam 1.5% were included in the analysis. Dermal tolerability assessment was evaluated based on the severity of skin reaction as mild, moderate, and severe. Incidence of moderate erythema reaction to minocycline foam 1.5% was significant [RR = 0.77, 95% CI = (0.62, 0.96), *P* = 0.02, I2 = 0%], while the incidence of mild and severe erythema were insignificant [RR = 0.95, 95% CI = (0.78, 1.15), *P* = 0.59, I2 = 43%] [RR = 0.51, 95% CI = (0.16, 1.16), *P* = 0.25, I2 = 0%], respectively (Supplementary Figure 2). There was no significant incidence of telangiectasia [RR = 1.02, 95% CI = (0.94, 1.11), *P* = 0.36, I2 = 0%] (Supplementary Figure 3), Burning/stinging [RR = 0.99, 95% CI = (0.62, 1.56), *P* = 0.95, I2 = 53%%] (Supplementary Figure 4), Flushing/blushing [RR = 1.07, 95% CI = (0.71, 1.60), *P* = 0.75, I2 = 77%] (Supplementary Figure 5), Dryness/xerosis [RR = 0.89, 95% CI = (0.74, 1.06), *P* = 0.20, I2 = 0%] (Supplementary Figure 6), Itching [RR = 0.97, 95% CI = (0.79, 1.20), *P* = 0.79, I2 = 0%] (Supplementary Figure 7), Peeling/desquamation [RR = 0.85, 95% CI = (0.70, 1.03), *P* = 0.10, I2 = 0%] (Supplementary Figure 8), and Hyperpigmentation [RR = 0.88, 95% CI = (0.68, 1.13), *P* = 0.30, I2 = 19%] (Supplementary Figure 9). Dermal tolerability assessment was rated as high in grade criteria except for burning and flushing which were moderate (Supplementary Table 1).

## Discussion

The results of our meta-analysis demonstrated that minocycline foam, particularly the 1.5% formulation, was effective in reducing inflammatory lesion counts compared to the vehicle. Both minocycline foam 1.5% and minocycline gel (1% and 3%) showed improvement in Investigator’s Global Assessment (IGA) scores at week 12, with minocycline foam outperforming the gel formulations in treatment success. Additionally, minocycline foam 1.5% showed greater efficacy in reducing inflammatory lesion counts compared to the vehicle. These findings suggest that minocycline foam, especially at 1.5%, offers a promising treatment for inflammatory skin conditions.

Our meta-analysis exhibited significant results regarding IGA score indicating higher treatment success among patients treated with FMX103. Additionally, both concentrations of minocycline gel (1% and 3%) did portray significant changes in IGA scores compared to vehicle preparation. These results are in line with the statements of Shaheen et al in a network meta-analysis. On the contrary, a multi-center clinical trial concluded that minocycline gel 1% did not have a significant IGA score reduction, while the 3% preparation had a significant outcome in this regard (3). The non-failure of minocycline gel preparations to achieve a reduction in IGA score may be explained by the properties of the gel itself hindering the absorption of the antibiotic in the skin. Further clinical trials are needed to properly assess the efficacy of minocycline gel. Our research findings demonstrated compelling evidence supporting a notable decrease in inflammatory lesions among patients treated with minocycline gel and foam formulations.

Minocycline gel (1% and 3%) demonstrated superior efficacy compared to the vehicle in treating moderate to severe papulopustular rosacea. Likewise, FMX103 1.5% achieved significant improvement over the vehicle at week 12 in regard to the absolute change from baseline in inflammatory lesions count further delineating the efficacy of topical minocycline preparations. This effect could be explained by the anti-inflammatory properties of minocycline though a network meta-analysis assessing the efficacy of antibiotics in rosacea recommended the use of systemic minocycline, in the absence of contraindications, as it was superior to minocycline foam (10). In our assessment of the safety characteristics within this meta-analysis, which included examining erythema, telangiectasia, itching, hyperpigmentation, flushing, peeling, dryness, and burning sensations, no notable outcomes were identified. These findings align with the recently publish studies, indicating that topical minocycline is well-tolerated in terms of adverse effects related to moderate to severe papulopustular rosacea (13, 14).

This study conducted a robust pooled analysis on the efficacy of topical minocycline preparations for treating moderate to severe papulopustular rosacea, offering valuable insights for clinical practice. However, several limitations need to be acknowledged. The presence of significant heterogeneity among the included studies, likely due to the limited number of studies available, is a notable constraint. Variability in the inclusion of key clinical outcomes like inflammatory lesion count, RosaQoL score, facial local tolerability, and patient satisfaction across different studies represent another limitation. Also, according to the Cochrane Collaboration guidelines, the assessment of publication bias is recommended only when the total number of studies included in a meta-analysis is 10 or more. As our analysis included fewer than 10 studies, a formal evaluation of publication bias was not conducted (15).

Additionally, the exclusion of the 3% minocycline foam from this study is a further constraint. Lastly, it is important to note that recent network meta-analyses comparing various treatment options for rosacea, including topical treatments, have been published, indicating the evolving landscape of research in this area.

Based on the findings of this study, it is recommended that topical minocycline preparations, particularly Minocycline 1.5% foam, be considered as effective treatment options for moderates to severe papulopustular rosacea. While other symptom indicators did not exhibit significant changes for either foam or gel formulations, the overall efficacy and safety profile of topical minocycline suggests its value in managing papulopustular rosacea. Further research and clinical trials are warranted to explore the full potential and optimal use of topical minocycline in the management of rosacea, addressing limitations such as heterogeneity among studies and the needs for comprehensive assessment of clinical outcomes.

## Conclusion

In conclusion, topical minocycline, particularly the 1.5% foam, demonstrated potential efficacy in reducing inflammatory lesion counts and improving IGA scores in moderate to severe papulopustular rosacea. However, the findings should be interpreted with caution due to statistical and clinical heterogeneity, as well as the low number of studies included in the analysis. The treatment exhibited a generally favorable safety profile, with minimal adverse effects such as moderate erythema. These results suggest that minocycline foam could be a promising therapeutic option, though further well-powered studies are needed to confirm its efficacy and safety in a broader patient population.

## Data Availability

The original contributions presented in this study are included in the article/Supplementary material, further inquiries can be directed to the corresponding author.
