# Supplementary material for: Efficacy and safety of topical minocycline preparations for papulopustular rosacea: a systematic review and meta-analysis
Source: Front Med (Lausanne). 2025 Apr 1;12:1517825. doi: 10.3389/fmed.2025.1517825 (PMC11996761; doi:10.3389/fmed.2025.1517825)
Supplement: Supplementary file 1 [file Data_Sheet_1.pdf]

Efficacy and Safety of Topical Minocycline Preparations for the Treatment of Moderate to Severe Papulopustular Rosacea; a Systematic Review and Meta-analysis.

Awadh Alamri[1,3] Abdulrahman H. Alsamadani[1,2], Rose A. Alraddadi[1,2], Mulham Kalantan[1,2], Randa Khafaji[2,3], Bader Bashrahil[1,2], Hassan Bogari[1,2], Athoub Kadasa[1,2], Abdulhadi Jfri[1,2,3]

[1]College of Medicine, King Saud Bin Abdulaziz University for Health Sciences, Jeddah, Saudi Arabia
[2]King Abdullah International Medical Research Center, Jeddah, Saudi Arabia.
[3]Division of Dermatology, Department of Medicine, Ministry of the National Guard-Health Affairs, Jeddah, Saudi Arabia.

Corresponding author:
Name: Dr. Awadh Mohammed Alamri
Address: Batha Quraysh Dist., Makkah, Saudi Arabia

Affiliation:

- King Saud bin Abdulaziz University for Health Sciences, Jeddah, Saudi Arabia.
- Division of Dermatology, Department of Medicine, Ministry of the National Guard-Health Affairs, Jeddah, Saudi Arabia.

Contact information:

- alamriaw@hotmail.com
- +966 55 555 0641

ORCID ID: 0009-0008-9874-9322

Figures Legend:

Table S1: The Grading of Recommendations Assessment, Development, and Evaluation (GRADE).

Table S2: Fund and endpoints of efficacy and safety.

Table S3: Results for each study.

Figure S1: Absolute change in the inflammatory lesion count

Figure S2: Erythema

Figure S3: Telangiectasia

Figure S4: Burning/stinging

Figure S5: Flushing/blushing

Figure S6: Dryness/xerosis

Figure S7: Itching

Figure S8: Peeling/desquamation

Figure S9: Hyperpigmintation

	Certainty assessment						Certainty
Outcome	Study design	Risk of bias	Inconsistency	Indirectness	Imprecision	Other considerations	
**change in inflammatory lesions count from baseline**	randomised trials	not serious	Not serious	not serious	serious[a]	none	⊕⊕⊕◯ Moderate

	Certainty assessment						
Outcome	Study design	Risk of bias	Inconsistency	Indirectness	Imprecision	Other considerations	Certainty
**IGA Score**	randomised trials	not serious	Not serious	not serious	not serious	none	⊕⊕⊕⊕ High
**Absolute change in the inflammatory lesions count**	randomised trials	not serious	serious[b]	not serious	serious[a]	none	⊕⊕⊕◯ Moderate
**Erythema**	randomised trials	not serious	not serious	not serious	not serious	none	⊕⊕⊕⊕ High
**Telangiectasia**	randomised trials	not serious	not serious	not serious	not serious	none	⊕⊕⊕⊕ High
**Burning/Stinging**	randomised trials	not serious	serious[b]	not serious	not serious	none	⊕⊕⊕◯ Moderate
**Flushing/Blushing**	randomised trials	not serious	serious[b]	not serious	not serious	none	⊕⊕⊕◯ Moderate
**Dryness/Xerosis**	randomised trials	not serious	not serious	not serious	not serious	none	⊕⊕⊕⊕ High
**Itching**	randomised trials	not serious	not serious	not serious	not serious	none	⊕⊕⊕⊕ High

	Certainty assessment						Certainty
Outcome	Study design	Risk of bias	Inconsistency	Indirectness	Imprecision	Other considerations	
**Peeling/Desquamation**	randomised trials	not serious	not serious	not serious	not serious	none	⊕⊕⊕⊕ High
**Hyperpigmentation**	randomised trials	not serious	not serious	not serious	not serious	none	⊕⊕⊕⊕ High

Explanations

a. Wide confidence interval
b. High heterogeneity

Table S1: The Grading of Recommendations Assessment, Development, and Evaluation (GRADE)

**Study**	**Safety**	**Efficacy**	**Fund**
NCT03263273	Standard safety assessments for adverse events were included at each study visit. Vital signs assessments and physical examinations were performed at each visit. Pregnancy tests were conducted on women of childbearing potential at baseline, week 6 and week 12. The investigator was asked to complete a local application site reaction scale to record application site reactions, including erythema, dryness, erosion/oedema and a skin discoloration score (on a scale of 0 to 6). At each visit, patients were asked	The primary outcome measure was the absolute mean reduction in inflammatory lesion count at week 12 when compared with baseline. Key secondary outcomes were the proportion of patients achieving assessments of 'clear' or 'almost clear' together with a two-grade reduction in the Investigator's Global Assessment (IGA) score at week 12, and the proportion of patients achieving 'clear' or 'almost clear' together with a two-grade reduction in the IGAe score at week 12. Independent assessments of erythema and	This study was sponsored and funded by Hovione Scientia Ltd. The founders had input into the design of the study. Conduct of the study, collection of data and analysis of the data were completed by a third-party contract research organization, to minimize bias. G.N.M. has had full access to all the data in the study and takes responsibility for the integrity of the data and the accuracy of the data analysis.

	whether they liked the product.	telangiectasia on 4-point scales were included. Investigators were trained during a live training session and provided with online resources and example booklets. Key assessments were completed by board-certified dermatologists, and the investigator assessing the patient at baseline was asked to complete all follow-up assessments for the patient.	
NCT03142451	Safety evaluations included adverse events (AEs), vital signs, physical examination, laboratory investigations, and local tolerability assessment.	The coprimary efficacy endpoints were the absolute change from baseline to week 12 in the inflammatory lesion count and the proportion of participants achieving endpoint success, defined as a dichotomized (yes/no) IGA score of 0 or 1 and at least a 2-grade improvement from baseline at week 12. The 5-point IGA scale (0, clear; 1, almost clear; 2, mild; 3, moderate; 4, severe) was based on the severity of the rosa cea, as indicated by the presence or absence of inflammatory papules, pustules, or nodules. Secondary endpoints included the dichotomized IGA score for endpoint success (where success was defined as a 2-grade improvement in score at week 12 compared with baseline), the abso lute change from baseline in inflammatory lesion count at week 4 andweek 8, and the percent change in the number of inflammatory lesions from baseline to week 12.	Supported by Foamix Pharmaceuticals, Inc.

Gold et al.	Safety and efficacy evaluations occurred during visits at Weeks 16, 22, 28, 34, 40, 46, and 52 of the open label extension study, relative to the baseline visit of the preceding double-blind study. Safety assessments included treatment-emergent adverse events (TEAEs), laboratory tests, vital signs, physical examinations, and local skin tolerability assessments. The coprimary efficacy endpoints were the absolute change in inflammatory lesion count at Week 52 compared to the baseline of the preceding double-blind study, and the IGA treatment success rate at Week 52, where success was defined as an IGA score of 0 or 1, and at least a two-grade improvement (reduction) from baseline. The absolute and percent change from baseline in inflammatory lesions and the dichotomized IGA treatment success rate at each visit were among the secondary efficacy endpoints. The Subject Satisfaction Questionnaire (SSQ) was administered at Week 52 as an additional efficacy endpoint.		This study was sponsored by Foamix Pharmaceuticals Ltd., a wholly owned subsidiary of VYNE Therapeutics Inc.
NCT02601963	Safety assessments were the standard safety measures, which included physical examinations, vital signs, adverse events, and clinical laboratory test results. Local safety assessments were also performed to measure the severity of signs and symptoms, including burning/stinging, flushing/nblushing, and telangiectasia.	The primary endpoint was the absolute change in inflammatory lesion counts at week 12 from baseline. Secondary endpoints included the proportion of subjects with improvement in IGA scores of at least two grades, the proportion who reached a score of ‘‘clear’’ or‘‘almost clear’’ (IGA 0/1), and the percent change in inflammatory lesion count at week 12. Clinical erythema and the Rosacea Quality of Life Index (RosaQoL) were also evaluated. Erythema of the face was graded at each study visit using a five-point scale as follows: 0 = clear skin with no signs of erythema; 1 = almost clear of erythema, slight redness; 2 = mild erythema, definite redness; 3 = moderate erythema, marked redness; and 4 = severe	This study was funded by Foamix Pharmaceuticals.

		erythema, fiery redness. The RosaQoL is a self-adminis tered questionnaire used to measure the health-related quality of life of patients who have rosacea, as described previously. The RosaQoL was collected at baseline and at week 12.	

Table S2: Fund and endpoints of efficacy and safety.

Study	Efficacy	Safety
NCT03263273	The study evaluated the efficacy and safety of minocycline 1% and 3% gels in reducing inflammatory lesions in comparison to a vehicle arm. At week 12, both minocycline arms showed significantly greater reductions in lesion counts (12.6 and 13.1, respectively) than the vehicle arm (7.9). Minocycline 3% demonstrated sustained efficacy at week 4 post-treatment cessation, while minocycline 1% did not. Investigator's Global Assessment (IGA) success rates at week 12 were 39% and 46% for minocycline 1% and 3%, respectively, versus 31% for the vehicle. Although the IGA including erythema (IGAe) showed no statistical differences, the minocycline 3% arm achieved a significant reduction in erythema severity (P = 0.039). Patient satisfaction was high, with 70%-72% favoring minocycline over the vehicle (49%). Treatment-related adverse events were mild and transient, and completion rates were comparable across arms. Overall, minocycline gels were effective, safe, and well-tolerated.	
NCT03142451	FMX103 1.5% demonstrated significant efficacy in treating rosacea across two studies, meeting both coprimary endpoints at week 12 by significantly reducing inflammatory lesion counts (Study 11: -17.57 vs -15.65, P = .0031; Study 12: -18.54 vs -14.88, P < .0001) and achieving higher Investigator's Global Assessment (IGA) treatment success rates (Study 11: 52.1% vs 43.0%, P = .0273; Study 12: 49.1% vs 39.0%, P = .0077). Secondary analyses showed reductions in inflammatory lesions as early as week 4, with continued improvement through week 12, including significant percent changes (e.g., Study 12: -40.3% vs -28.5%, P < .0001). FMX103 1.5% also achieved superior IGA endpoint success at week 12 (Study 11: 55.3% vs 45.8%,	FMX103 1.5% demonstrated favorable safety and tolerability over 12 weeks of daily application, with no serious treatment-emergent adverse events (TEAEs) reported in either study. Discontinuation rates were low and comparable between the FMX103 and vehicle groups, with one participant in Study 12 discontinuing due to pruritus related to FMX103. Noncutaneous TEAEs, such as viral upper respiratory tract infections, upper respiratory tract infections, and headaches, were mild or moderate and occurred at similar rates between FMX103 and vehicle groups. Cutaneous TEAEs were rare (<1%), with pruritus being the most common. Over 95% of participants experienced no or only mild skin tolerability issues, and more than 80% reported no or mild telangiectasia or erythema at the

	P = .0171; Study 12: 53.8% vs 45.1%, P = .0189), with high patient satisfaction and improvement in global assessments.	application site. The proportion of participants rated as clear or almost clear for erythema significantly increased from baseline (4.3%-6.6%) to week 12 (36.4%-48.3%).
Gold et al.	Long-term treatment with FMX103 1.5% demonstrated sustained efficacy, with a progressive reduction in inflammatory lesions and increased rates of IGA treatment success over 52 weeks. The mean absolute change in inflammatory lesions from the double-blind baseline to Week 52 was –22.8 lesions across the pooled population, with comparable decreases in the FMX103/FMX103 group (−23.0 lesions) and the vehicle/FMX103 group (−22.5 lesions). The mean percentage change in inflammatory lesions at Week 52 was −82.3% overall, with similar reductions in both groups (FMX103/FMX103: −83.01%; vehicle/FMX103: −80.91%). Additionally, 79.8% of participants achieved IGA treatment success at Week 52, indicating robust and consistent long-term efficacy.	Long-term use of FMX103 1.5% minocycline foam was generally safe and well tolerated over 52 weeks, with low incidences of treatment-emergent adverse events (TEAEs) and treatment-related TEAEs. TEAEs occurred in 41.3% of the FMX103/FMX103 group and 37.2% of the vehicle/FMX103 group, with upper respiratory infections and sinusitis being the most common. Serious TEAEs were infrequent (1.8% in FMX103/FMX103 and 2.3% in vehicle/FMX103), and none were treatment-related. Most TEAEs were mild or moderate, with only one severe case of pruritus likely related to treatment. Local facial signs and symptoms such as erythema, dryness, and burning significantly improved over time, with >57% of participants showing no signs of adverse skin reactions by Week 52. The proportion of subjects with clear or almost clear erythema increased markedly from baseline (4.0%) to the end of the open-label study (59.3%), demonstrating both the safety and tolerability of long-term treatment.
NCT02601963	At Week 12, FMX103 (1.5% and 3%) demonstrated superior efficacy over vehicle foam in reducing inflammatory lesions, with mean reductions of 21.1 and 19.9 lesions, respectively, compared to 7.8 lesions for vehicle (p < 0.001). Corresponding percentage reductions were 61.4% for 1.5% FMX103 and 55.5% for 3%, versus 29.7% for vehicle (p < 0.001). Improvements in IGA scores were significantly greater in FMX103 groups, with 41.8% (1.5%) and 33.3% (3%) achieving at least a two-grade improvement, compared to 17.9% for vehicle (p = 0.002 and p = 0.032, respectively). Additionally, 25.3% (1.5%) and 17.3% (3%) achieved "clear" or	Both doses of FMX103 (1.5% and 3%) were safe and well-tolerated, with 47% of subjects reporting at least one treatment-emergent adverse event (TEAE), most commonly nasopharyngitis (10%) and urinary tract infection (3%). Treatment-related TEAEs, reported by 4.7% of subjects, were primarily mild dermal reactions, resolving by the study's end, with no systemic TEAEs reported. Serious TEAEs, including contusion, cerebral hemorrhage, and hemorrhoids, occurred in isolated cases across groups but were unrelated to treatment. Four subjects discontinued due to dermal-related TEAEs, all of which resolved. Overall, FMX103 was well-

	"almost clear" IGA scores versus 7.7% for vehicle (p = 0.001 and p = 0.041). Erythema improved notably, with 76% (1.5%) and 85% (3%) achieving "clear" to "mild" ratings, compared to 68% for vehicle, alongside significant health-related quality of life improvements.	tolerated, with comparable severity of local symptoms across treatment groups.

Table S3: Results for each study.

Study	Change in inflammatory lesion count	IGA treatment success (1.5%)	Absolute change in the inflammatory lesion count (1.5%)	Adverse events
NCT03142451	Minocycline (64.13 ±1.58) Placebo (56.62 ±2.13)	Minocycline (258/495), Placebo (111/256)	Minocycline (17.56 ±9.8) Placebo (15.3±9.6)	No significant difference between the groups.
NCT04608500	Minocycline (61.45 ±1.57) Placebo (50.16 ±2.22)	Minocycline (252/514), Placebo (100/257)	Minocycline (18.38 ±0.5) Placebo (14.5±0.7)	No significant difference between the groups.
NCT02601963	-	Minocycline (20/79), Placebo (6/79)	-	-
NCT03263273	-	1%: Minocycline (35/90), Placebo (24/78)	1%: Minocycline (12.6 ±2.4) Placebo (8.9±10.8)	-

Table S4: Main results for each study.

Figures:

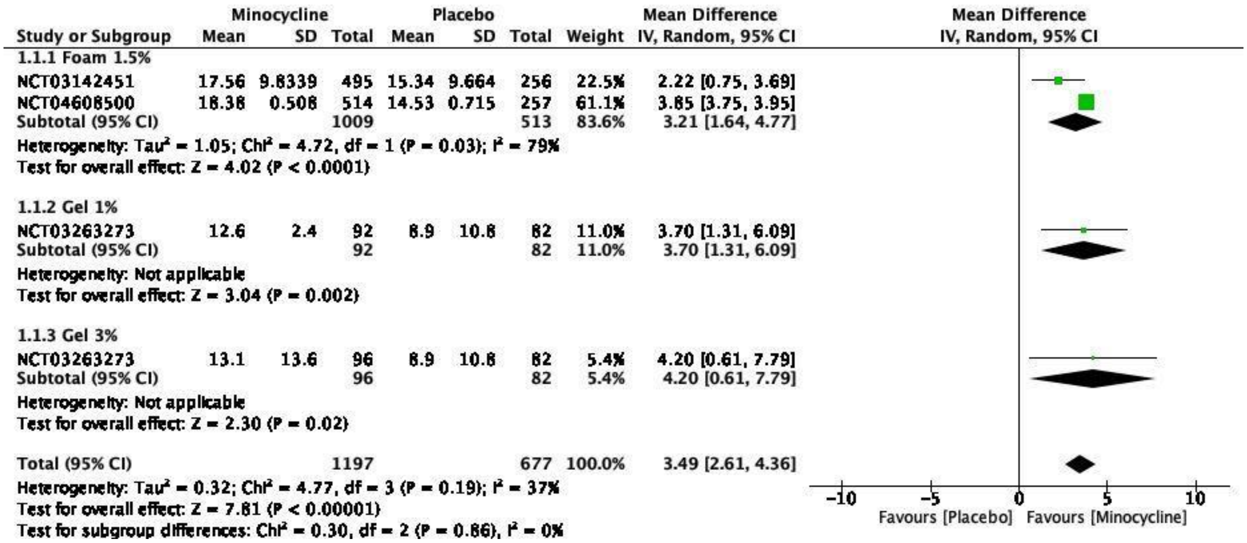

Figure S1: Absolute change in the inflammatory lesion count

Study or Subgroup	Minocycline Events	Minocycline Total	Placebo Events	Placebo Total	Weight	Risk Ratio IV, Random, 95% CI
**1.6.1 Mild**						
NCT03142451	169	423	86	225	33.3%	1.05 [0.85, 1.28]
NCT04608500	156	474	90	235	32.3%	0.86 [0.70, 1.06]
Subtotal (95% CI)		897		460	65.6%	0.95 [0.78, 1.15]
Total events	325		176			
Heterogeneity: Tau² = 0.01; Chi² = 1.75, df = 1 (P = 0.19); I² = 43%						
Test for overall effect: Z = 0.54 (P = 0.59)						
**1.6.2 Moderate**						
NCT03142451	80	423	55	225	16.6%	0.77 [0.57, 1.05]
NCT04608500	84	474	54	235	16.5%	0.77 [0.57, 1.05]
Subtotal (95% CI)		897		460	33.1%	0.77 [0.62, 0.96]
Total events	164		109			
Heterogeneity: Tau² = 0.00; Chi² = 0.00, df = 1 (P = 0.99); I² = 0%						
Test for overall effect: Z = 2.36 (P = 0.02)						
**1.6.3 Severe**						
NCT03142451	1	423	2	225	0.3%	0.27 [0.02, 2.92]
NCT04608500	5	474	4	235	1.0%	0.62 [0.17, 2.29]
Subtotal (95% CI)		897		460	1.3%	0.51 [0.16, 1.61]
Total events	6		6			
Heterogeneity: Tau² = 0.00; Chi² = 0.37, df = 1 (P = 0.54); I² = 0%						
Test for overall effect: Z = 1.15 (P = 0.25)						
**Total (95% CI)**		2691		1380	100.0%	0.88 [0.77, 1.00]
Total events	495		291			
Heterogeneity: Tau² = 0.00; Chi² = 5.46, df = 5 (P = 0.36); I² = 8%						
Test for overall effect: Z = 1.94 (P = 0.05)						
Test for subgroup differences: Chi² = 2.76, df = 2 (P = 0.25), I² = 27.6%						

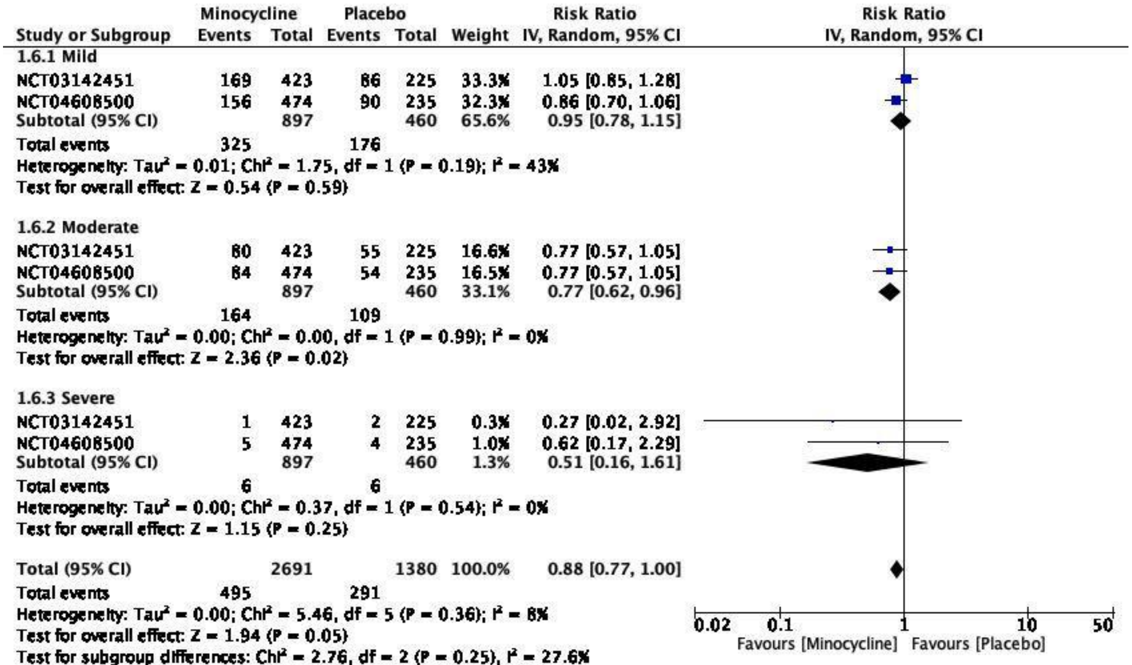

Figure S2: Erythema

Study or Subgroup	Minocycline Events	Minocycline Total	Placebo Events	Placebo Total	Weight	Risk Ratio IV, Random, 95% CI
**1.7.1 Mild**						
NCT03142451	280	423	146	225	51.4%	1.02 [0.91, 1.15]
NCT04608500	267	474	126	235	34.9%	1.05 [0.91, 1.21]
Subtotal (95% CI)		897		460	86.4%	1.03 [0.94, 1.13]
Total events	547		272			
Heterogeneity: Tau² = 0.00; Chi² = 0.10, df = 1 (P = 0.76); I² = 0%						
Test for overall effect: Z = 0.69 (P = 0.49)						
**1.7.2 Moderate**						
NCT03142451	78	423	43	225	6.3%	0.96 [0.69, 1.35]
NCT04608500	91	474	48	235	7.3%	0.94 [0.69, 1.28]
Subtotal (95% CI)		897		460	13.6%	0.95 [0.76, 1.20]
Total events	169		91			
Heterogeneity: Tau² = 0.00; Chi² = 0.01, df = 1 (P = 0.91); I² = 0%						
Test for overall effect: Z = 0.43 (P = 0.67)						
**1.7.3 Severe**						
NCT03142451	0	423	0	225		Not estimable
NCT04608500	0	474	0	235		Not estimable
Subtotal (95% CI)		897		460		Not estimable
Total events	0		0			
Heterogeneity: Not applicable						
Test for overall effect: Not applicable						
**Total (95% CI)**		2691		1380	100.0%	1.02 [0.94, 1.11]
Total events	716		363			
Heterogeneity: Tau² = 0.00; Chi² = 0.53, df = 3 (P = 0.91); I² = 0%						
Test for overall effect: Z = 0.48 (P = 0.63)						
Test for subgroup differences: Chi² = 0.42, df = 1 (P = 0.52), I² = 0%						

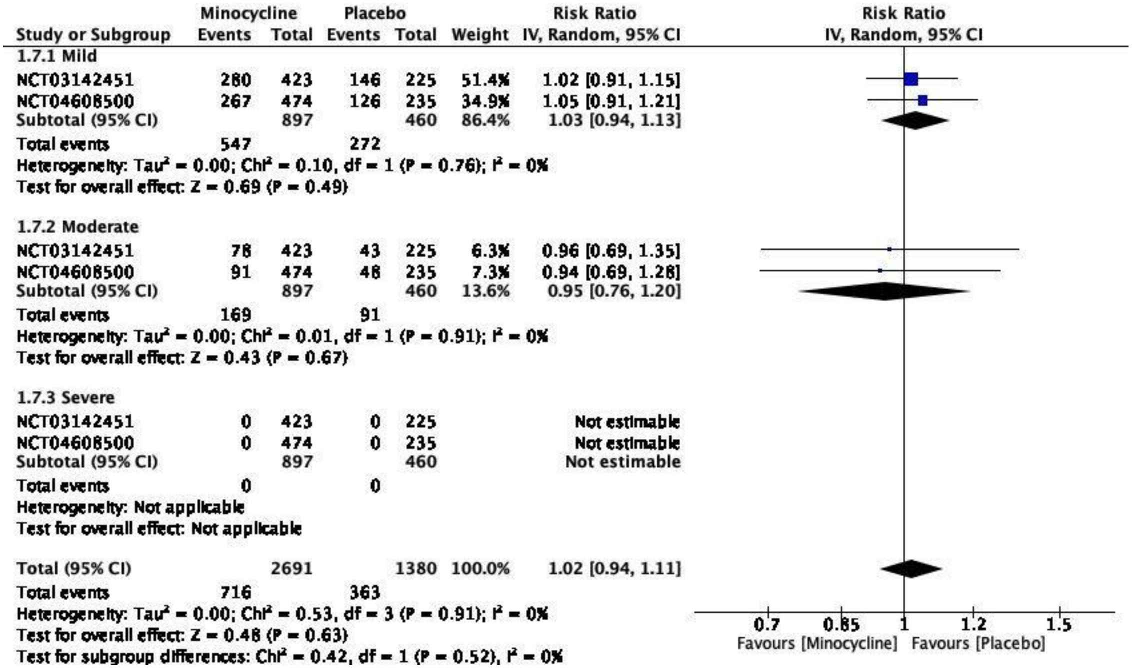

Figure S3: Telangiectasia

Study or Subgroup	Minocycline Events	Minocycline Total	Placebo Events	Placebo Total	Weight	Risk Ratio IV, Random, 95% CI
**1.9.1 Mild**						
NCT03142451	71	423	39	225	40.7%	0.97 [0.68, 1.38]
NCT04608500	48	474	36	235	38.1%	0.66 [0.44, 0.99]
Subtotal (95% CI)		897		460	78.8%	0.81 [0.56, 1.18]
Total events	119		75			
Heterogeneity: Tau² = 0.04; Chi² = 1.94, df = 1 (P = 0.16); I² = 48%						
Test for overall effect: Z = 1.11 (P = 0.27)						
**1.9.2 Moderate**						
NCT03142451	16	423	3	225	11.1%	2.84 [0.84, 9.63]
NCT04608500	9	474	3	235	10.1%	1.49 [0.41, 5.44]
Subtotal (95% CI)		897		460	21.2%	2.09 [0.86, 5.10]
Total events	25		6			
Heterogeneity: Tau² = 0.00; Chi² = 0.50, df = 1 (P = 0.48); I² = 0%						
Test for overall effect: Z = 1.63 (P = 0.10)						
**1.9.3 Severe**						
NCT03142451	0	423	0	225		Not estimable
NCT04608500	0	474	0	235		Not estimable
Subtotal (95% CI)		897		460		Not estimable
Total events	0		0			
Heterogeneity: Not applicable						
Test for overall effect: Not applicable						
**Total (95% CI)**		2691		1380	100.0%	0.99 [0.62, 1.56]
Total events	144		81			
Heterogeneity: Tau² = 0.10; Chi² = 6.36, df = 3 (P = 0.10); I² = 53%						
Test for overall effect: Z = 0.06 (P = 0.95)						
Test for subgroup differences: Chi² = 3.72, df = 1 (P = 0.05), I² = 73.1%						

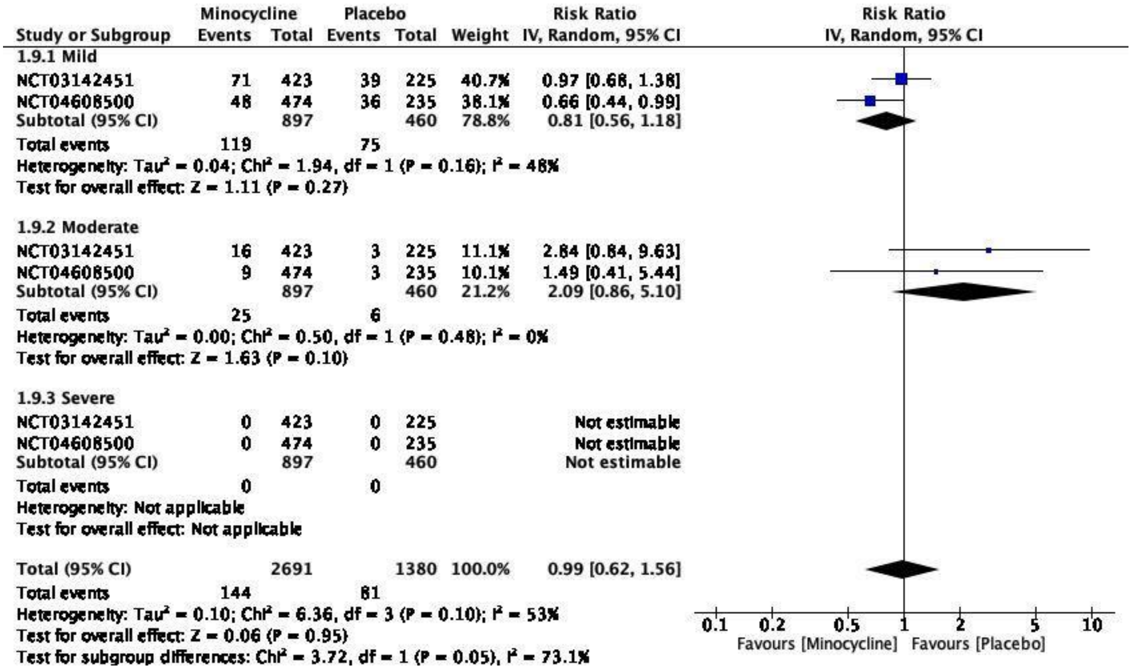

Figure S4: Burning/stinging

Study or Subgroup	Minocycline Events	Minocycline Total	Placebo Events	Placebo Total	Weight	Risk Ratio IV, Random, 95% CI
**1.10.1 Mild**						
NCT03142451	186	423	94	225	31.5%	1.05 [0.87, 1.27]
NCT04608500	164	474	104	235	31.4%	0.78 [0.65, 0.94]
Subtotal (95% CI)		897		460	62.9%	0.91 [0.68, 1.21]
Total events	350		198			
Heterogeneity: Tau² = 0.03; Chi² = 4.76, df = 1 (P = 0.03); I² = 79%						
Test for overall effect: Z = 0.65 (P = 0.51)						
**1.10.2 Moderate**						
NCT03142451	36	423	20	225	21.7%	0.96 [0.57, 1.61]
NCT04608500	50	474	2	235	6.7%	12.39 [3.04, 50.50]
Subtotal (95% CI)		897		460	28.3%	3.16 [0.26, 38.64]
Total events	86		22			
Heterogeneity: Tau² = 2.99; Chi² = 11.22, df = 1 (P = 0.0008); I² = 91%						
Test for overall effect: Z = 0.90 (P = 0.37)						
**1.10.3 Severe**						
NCT03142451	7	423	2	225	5.6%	1.86 [0.39, 8.89]
NCT04608500	1	474	4	235	3.1%	0.12 [0.01, 1.10]
Subtotal (95% CI)		897		460	8.7%	0.54 [0.04, 7.58]
Total events	8		6			
Heterogeneity: Tau² = 2.73; Chi² = 3.91, df = 1 (P = 0.05); I² = 74%						
Test for overall effect: Z = 0.46 (P = 0.65)						
**Total (95% CI)**		2691		1380	100.0%	1.07 [0.71, 1.60]
Total events	444		226			
Heterogeneity: Tau² = 0.13; Chi² = 21.98, df = 5 (P = 0.0005); I² = 77%						
Test for overall effect: Z = 0.33 (P = 0.75)						
Test for subgroup differences: Chi² = 1.10, df = 2 (P = 0.58), I² = 0%						

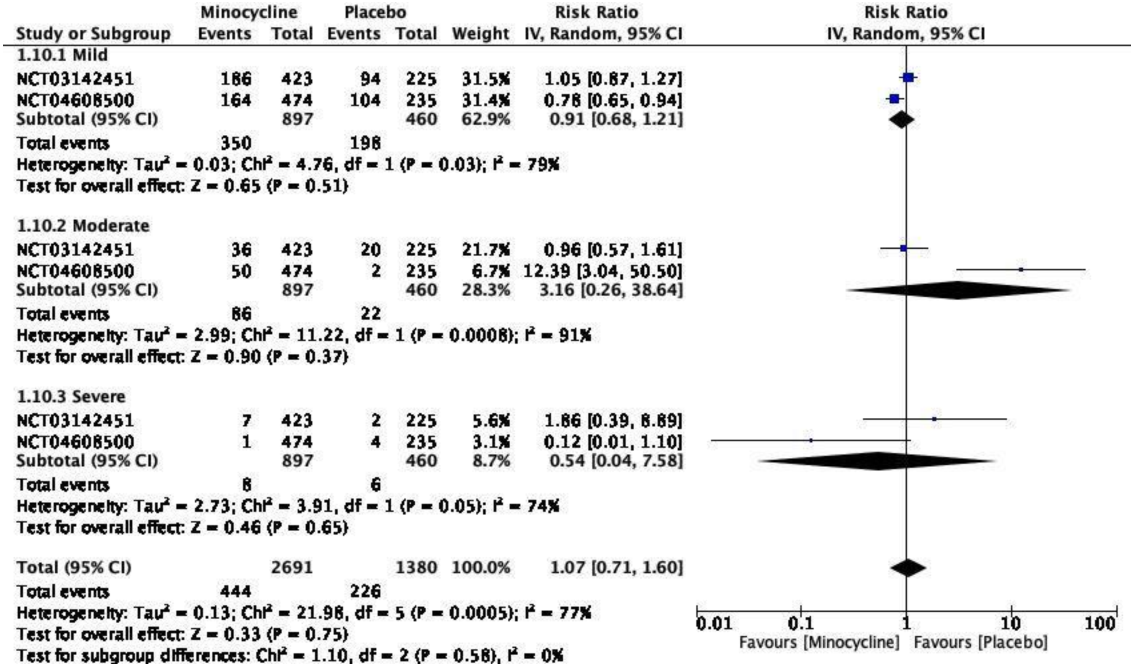

Figure S5: Flushing/blushing

Study or Subgroup	Minocycline Events	Minocycline Total	Placebo Events	Placebo Total	Weight	Risk Ratio IV, Random, 95% CI
**1.11.1 Mild**						
NCT03142451	107	423	66	225	47.4%	0.86 [0.66, 1.12]
NCT04608500	107	474	55	235	39.6%	0.96 [0.73, 1.28]
Subtotal (95% CI)		897		460	87.0%	0.91 [0.75, 1.10]
Total events	214		121			
Heterogeneity: Tau² = 0.00; Chi² = 0.32, df = 1 (P = 0.57); I² = 0%						
Test for overall effect: Z = 0.99 (P = 0.32)						
**1.11.2 Moderate**						
NCT03142451	15	423	11	225	5.6%	0.73 [0.34, 1.55]
NCT04608500	21	474	13	235	7.1%	0.80 [0.41, 1.57]
Subtotal (95% CI)		897		460	12.7%	0.77 [0.46, 1.27]
Total events	36		24			
Heterogeneity: Tau² = 0.00; Chi² = 0.04, df = 1 (P = 0.85); I² = 0%						
Test for overall effect: Z = 1.03 (P = 0.30)						
**1.11.3 Severe**						
NCT03142451	1	423	0	225	0.3%	1.60 [0.07, 39.09]
NCT04608500	0	474	0	235		Not estimable
Subtotal (95% CI)		897		460	0.3%	1.60 [0.07, 39.09]
Total events	1		0			
Heterogeneity: Not applicable						
Test for overall effect: Z = 0.29 (P = 0.77)						
**Total (95% CI)**		2691		1380	100.0%	0.89 [0.74, 1.06]
Total events	251		145			
Heterogeneity: Tau² = 0.00; Chi² = 0.86, df = 4 (P = 0.93); I² = 0%						
Test for overall effect: Z = 1.27 (P = 0.20)						
Test for subgroup differences: Chi² = 0.50, df = 2 (P = 0.78), I² = 0%						

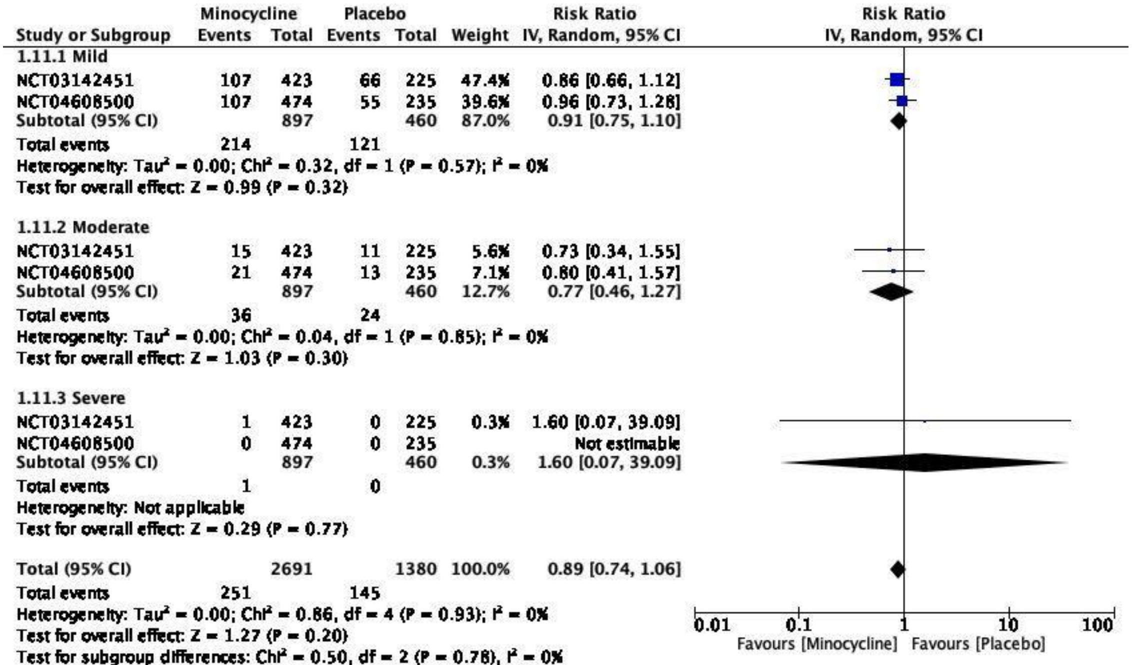

Figure S6: Dryness/xerosis

Study or Subgroup	Minocycline Events	Minocycline Total	Placebo Events	Placebo Total	Weight	Risk Ratio IV, Random, 95% CI
**1.12.1 Mild**						
NCT03142451	89	423	50	225	46.5%	0.95 [0.70, 1.29]
NCT04608500	90	474	45	235	42.1%	0.99 [0.72, 1.37]
Subtotal (95% CI)		897		460	88.5%	0.97 [0.78, 1.21]
Total events	179		95			
Heterogeneity: Tau² = 0.00; Chi² = 0.04, df = 1 (P = 0.84); I² = 0%						
Test for overall effect: Z = 0.29 (P = 0.77)						
**1.12.2 Moderate**						
NCT03142451	15	423	10	225	7.1%	0.80 [0.36, 1.75]
NCT04608500	15	474	5	235	4.4%	1.49 [0.55, 4.04]
Subtotal (95% CI)		897		460	11.5%	1.01 [0.55, 1.87]
Total events	30		15			
Heterogeneity: Tau² = 0.00; Chi² = 0.92, df = 1 (P = 0.34); I² = 0%						
Test for overall effect: Z = 0.04 (P = 0.97)						
**1.12.3 Severe**						
NCT03142451	0	423	0	225		Not estimable
NCT04608500	0	474	0	235		Not estimable
Subtotal (95% CI)		897		460		Not estimable
Total events	0		0			
Heterogeneity: Not applicable						
Test for overall effect: Not applicable						
**Total (95% CI)**		2691		1380	100.0%	0.97 [0.79, 1.20]
Total events	209		110			
Heterogeneity: Tau² = 0.00; Chi² = 0.98, df = 3 (P = 0.81); I² = 0%						
Test for overall effect: Z = 0.26 (P = 0.79)						
Test for subgroup differences: Chi² = 0.02, df = 1 (P = 0.90), I² = 0%						

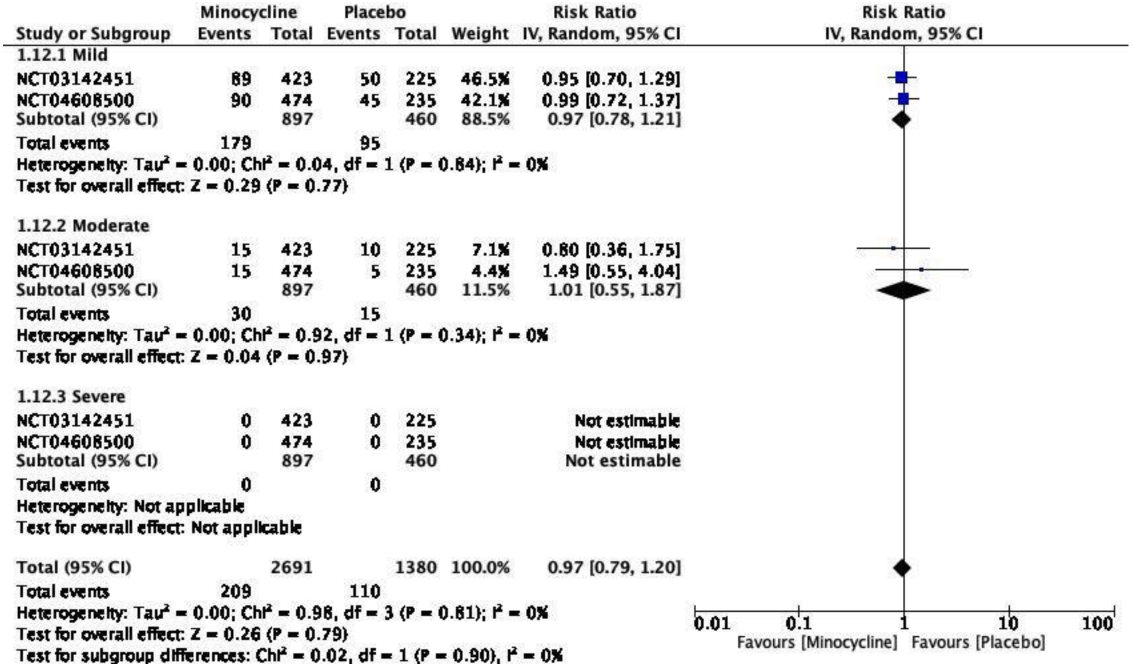

Figure S7: Itching

Study or Subgroup	Minocycline Events	Minocycline Total	Placebo Events	Placebo Total	Weight	Risk Ratio IV, Random, 95% CI
**1.13.1 Mild**						
NCT03142451	68	423	44	225	31.4%	0.82 [0.58, 1.16]
NCT04608500	76	474	44	235	32.6%	0.86 [0.61, 1.20]
Subtotal (95% CI)		897		460	63.9%	0.84 [0.66, 1.07]
Total events	144		88			
Heterogeneity: $Tau^2 = 0.00$; $Chi^2 = 0.03$, df = 1 (P = 0.87); $I^2 = 0\%$						
Test for overall effect: Z = 1.43 (P = 0.15)						
**1.13.2 Moderate**						
NCT03142451	9	423	5	225	3.2%	0.96 [0.32, 2.82]
NCT04608500	76	474	44	235	32.6%	0.86 [0.61, 1.20]
Subtotal (95% CI)		897		460	35.7%	0.86 [0.63, 1.19]
Total events	85		49			
Heterogeneity: $Tau^2 = 0.00$; $Chi^2 = 0.04$, df = 1 (P = 0.85); $I^2 = 0\%$						
Test for overall effect: Z = 0.89 (P = 0.38)						
**1.13.3 Severe**						
NCT03142451	1	423	0	225	0.4%	1.60 [0.07, 39.09]
NCT04608500	0	474	0	235		Not estimable
Subtotal (95% CI)		897		460	0.4%	1.60 [0.07, 39.09]
Total events	1		0			
Heterogeneity: Not applicable						
Test for overall effect: Z = 0.29 (P = 0.77)						
**Total (95% CI)**		2691		1380	100.0%	0.85 [0.70, 1.03]
Total events	230		137			
Heterogeneity: $Tau^2 = 0.00$; $Chi^2 = 0.24$, df = 4 (P = 0.99); $I^2 = 0\%$						
Test for overall effect: Z = 1.65 (P = 0.10)						
Test for subgroup differences: $Chi^2 = 0.17$, df = 2 (P = 0.92), $I^2 = 0\%$						

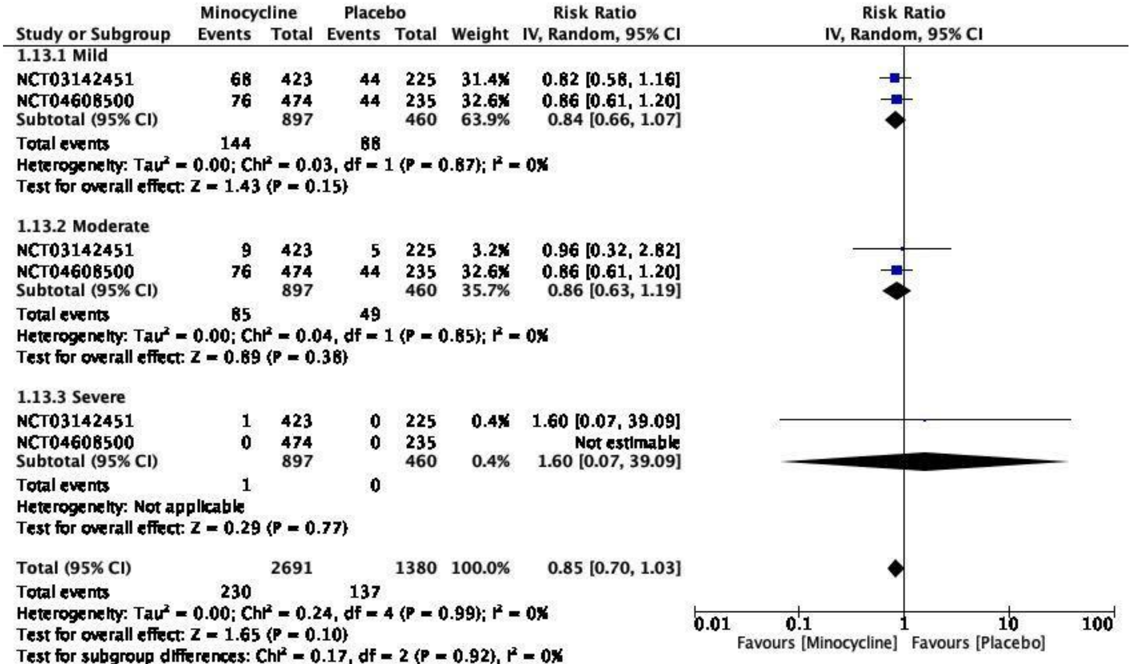

Figure S8: Peeling/desquamation

Study or Subgroup	Minocycline Events	Minocycline Total	Placebo Events	Placebo Total	Weight	Risk Ratio IV, Random, 95% CI
**1.14.1 Mild**						
NCT03142451	67	423	45	225	35.4%	0.79 [0.56, 1.11]
NCT04608500	135	474	76	235	54.0%	0.88 [0.70, 1.11]
Subtotal (95% CI)		897		460	89.4%	0.85 [0.70, 1.03]
Total events	202		121			
Heterogeneity: $Tau^2 = 0.00$; $Chi^2 = 0.25$, df = 1 (P = 0.61); $I^2 = 0\%$						
Test for overall effect: Z = 1.64 (P = 0.10)						
**1.14.2 Moderate**						
NCT03142451	18	423	4	225	5.3%	2.39 [0.82, 6.99]
NCT04608500	7	474	5	235	4.7%	0.69 [0.22, 2.16]
Subtotal (95% CI)		897		460	10.0%	1.31 [0.39, 4.40]
Total events	25		9			
Heterogeneity: $Tau^2 = 0.45$; $Chi^2 = 2.41$, df = 1 (P = 0.12); $I^2 = 59\%$						
Test for overall effect: Z = 0.43 (P = 0.66)						
**1.14.3 Severe**						
NCT03142451	0	423	0	225		Not estimable
NCT04608500	0	474	1	235	0.6%	0.17 [0.01, 4.05]
Subtotal (95% CI)		897		460	0.6%	0.17 [0.01, 4.05]
Total events	0		1			
Heterogeneity: Not applicable						
Test for overall effect: Z = 1.10 (P = 0.27)						
**Total (95% CI)**		2691		1380	100.0%	0.88 [0.68, 1.13]
Total events	227		131			
Heterogeneity: $Tau^2 = 0.02$; $Chi^2 = 4.92$, df = 4 (P = 0.30); $I^2 = 19\%$						
Test for overall effect: Z = 1.03 (P = 0.30)						
Test for subgroup differences: $Chi^2 = 1.49$, df = 2 (P = 0.48), $I^2 = 0\%$						

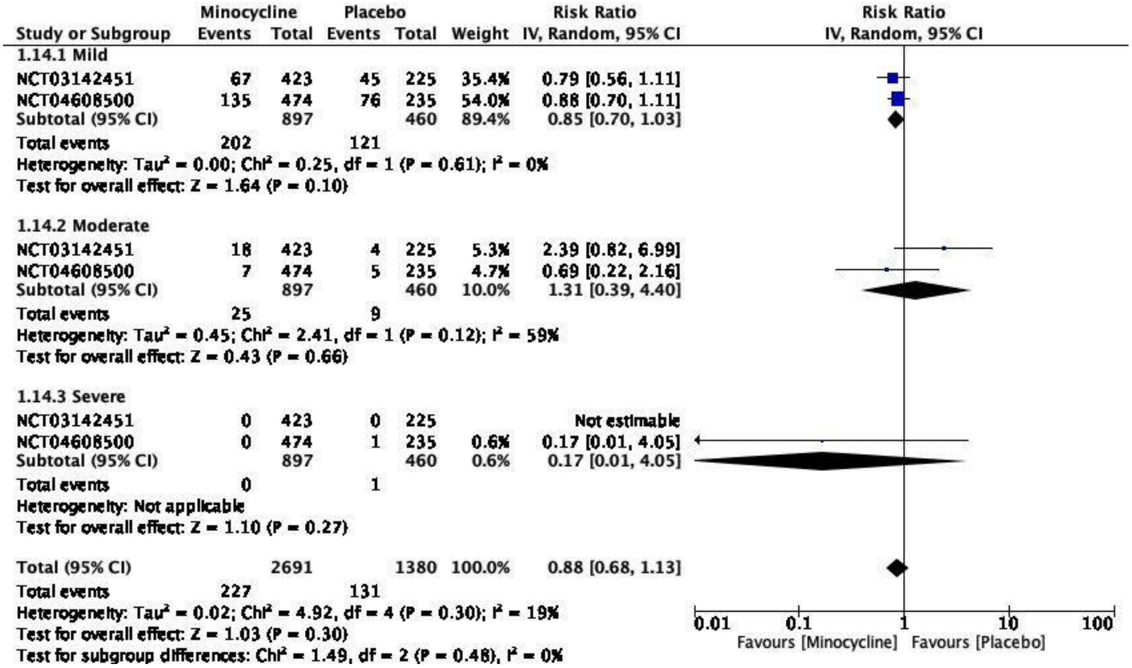

Figure S9: Hyperpigmentation

Search Strategy:

Keywords:
P: rosacea, papulopustular rosacea
I: topical minocycline, FMX103, zilxi, minocycline gel, minocycline foam, minocycline cream, minocycline, Versagel, Calumet
C: Vehicle foam
O: inflammatory lesions
D: RCT

papulopustular rosacea OR papulopustular OR rosacea
AND
topical minocycline OR FMX103 OR FMX103 1.5% OR FMX103 minocycline foam OR foam fmx103 OR zilxi OR minocycline foam OR minocycline OR minocycline 1.5% OR foam OR topical foam
AND
Randomized controlled trial OR Randomized trial OR Clinical trial OR trial OR RCT

Pubmed: (1652)
(((papulopustular rosacea) OR (papulopustular rosacea[MeSH Terms])) OR (papulopustular)) OR (papulopustular[MeSH Terms])) OR (rosacea)) OR (rosacea[MeSH Terms]))) AND ((((((topical minocycline) OR (topical minocycline[MeSH Terms])) OR (FMX103[MeSH Terms])) OR (FMX103)) OR (FMX103 1.5%)) OR (FMX103 1.5%[MeSH Terms])) OR (FMX103 minocycline foam[MeSH Terms])) OR (FMX103 minocycline foam)) OR (foam fmx103)) OR (foam fmx103[MeSH Terms])) OR (zilxi[MeSH Terms])) OR (zilxi)) OR (minocycline foam)) OR (minocycline foam[MeSH Terms])) OR (minocycline[MeSH Terms])) OR (minocycline)) OR (minocycline 1.5%)) OR (minocycline 1.5%[MeSH Terms])) OR (foam[MeSH Terms])) OR (foam)) OR (topical foam)) OR (topical foam[MeSH Terms])))) OR (FMX 3%)) OR (FMX103 3%)) OR ("minocycline gel"[All Fields])))) AND ((((((((((Randomized controlled trial) OR (Randomized controlled trial[MeSH Terms])) OR (Randomized trial[MeSH Terms])) OR (Randomized trial)) OR (Clinical trial)) OR (Clinical trial[MeSH Terms])) OR (trial[MeSH Terms])) OR (trial)) OR (RCT)) OR (RCT[MeSH Terms]))) Filters: Clinical Trial, Randomized Controlled Trial

SDL:
((((((papulopustular rosacea) OR (papulopustular rosacea[MeSH Terms])) OR (papulopustular)) OR (papulopustular[MeSH Terms])) OR (rosacea)) OR (rosacea[MeSH Terms])) AND ((((((((((((((((((((topical minocycline) OR (topical minocycline[MeSH Terms])) OR (FMX103[MeSH Terms])) OR (FMX103)) OR (FMX103 1.5%)) OR (FMX103 1.5%[MeSH Terms])) OR (FMX103 minocycline foam[MeSH Terms])) OR (FMX103 minocycline foam)) OR (foam fmx103)) OR (foam fmx103[MeSH Terms])) OR (zilxi[MeSH Terms])) OR (zilxi)) OR (minocycline foam)) OR (minocycline foam[MeSH Terms])) OR (minocycline[MeSH Terms])) OR (minocycline)) OR (minocycline 1.5%)) OR (minocycline 1.5%[MeSH Terms])) OR (foam[MeSH Terms])) OR (foam)) OR (topical foam)) OR (topical foam[MeSH Terms]))) AND (((((((((Randomized controlled trial) OR (Randomized controlled trial[MeSH Terms]))

OR (Randomized trial[MeSH Terms])) OR (Randomized trial)) OR (Clinical trial)) OR (Clinical trial[MeSH Terms])) OR (trial[MeSH Terms])) OR (trial)) OR (RCT)) OR (RCT[MeSH Terms]))

Cochrane Library: (132)

(("papulopustular rosacea") OR (papulopustular) OR ("rosacea")) AND ((topical minocycline) OR (FMX103) OR (FMX103 1.5%) OR (FMX103 minocycline foam) OR (zilxi) OR (minocycline foam) OR (minocycline) OR (minocycline 1.5%) OR (topical foam)) AND (("randomized controlled trial") OR ("randomized-controlled trials") OR ("RCT") OR ("clinical trial") OR (Randomized trial))
